# Discovering MoRFs by trisecting intrinsically disordered protein sequence into terminals and middle regions

**DOI:** 10.1186/s12859-018-2396-7

**Published:** 2019-02-04

**Authors:** Ronesh Sharma, Alok Sharma, Ashwini Patil, Tatsuhiko Tsunoda

**Affiliations:** 1 School of Engineering and Physics, The University of the South Pacific, Suva, Fiji; 2 School of Electrical and Electronics Engineering, Fiji National University, Suva, Fiji; 3 Laboratory of Medical Science Mathematics, RIKEN Center for Integrative Medical Sciences, Yokohama, 230-0045 Japan; 4 Department of Medical Science Mathematics, Medical Research Institute, Tokyo Medical and Dental University (TMDU), Tokyo, 113-8510 Japan; 5 Institute for Integrated and Intelligent Systems, Griffith University, Nathan, Brisbane, QLD Australia; 6 Human Genome Center, The Institute of Medical Science, The University of Tokyo, Tokyo, 108-8639 Japan; 7 CREST, JST, Tokyo, 113-8510 Japan

## Abstract

**Background:**

Molecular Recognition Features (MoRFs) are short protein regions present in intrinsically disordered protein (IDPs) sequences. MoRFs interact with structured partner protein and upon interaction, they undergo a disorder-to-order transition to perform various biological functions. Analyses of MoRFs are important towards understanding their function.

**Results:**

Performance is reported using the MoRF dataset that has been previously used to compare the other existing MoRF predictors. The performance obtained in this study is equivalent to the benchmarked OPAL predictor, i.e., OPAL achieved AUC of 0.815, whereas the model in this study achieved AUC of 0.819 using TEST set.

**Conclusion:**

Achieving comparable performance, the proposed method can be used as an alternative approach for MoRF prediction.

**Electronic supplementary material:**

The online version of this article (10.1186/s12859-018-2396-7) contains supplementary material, which is available to authorized users.

## Background

In the traditional view, the function of protein critically depends on the well-defined three-dimensional structure. This concept implies that protein sequence defines the structure, which in turn outlines the protein function. However, recent studies have revealed that many proteins do not form a defined three-dimensional structure but they are functional [1–4]. These proteins are called intrinsically disordered proteins (IDPs) or intrinsically disordered regions (IDRs). IDPs and IDRs lack the hydrophobic cores which makeup the structured domain. Thus, the functionality of these proteins arises in a different manner compared to the protein structure-function paradigm.

IDPs consist of functional sites that are associated with important cellular functions, such as transcriptional regulation and signal transduction [2, 3]. Molecular recognition features (MoRFs) are one of the important functional sites that reside in IDPs and they permit interaction with structured partner proteins [2, 5, 6]. Upon interaction, they undergo a disorder-to-order transition and adopt conformations such as α-helix (α-MoRFs), β-strand (β-MoRFs), and γ-coil (γ-MoRFs) or mixtures of these complex-MoRFs. For a deeper understanding of disordered proteins and MoRFs, several studies have been done and databases have been introduced [5–10].

Analyses of MoRFs can be done using experimental methods, however, these experiments are time-consuming and expensive to perform. Therefore, it is prudent to computationally identify MoRFs in disordered protein sequences. Many machine learning methods for predicting MoRFs have been studied [8, 9, 11–15] in this respect. A detailed literature review of the available state-of-the-art methods has been thoroughly done in our previous work [15].

Analyzing the structural properties of MoRFs, their conformational behavior, and their interaction mechanism with various binding region helps in the understanding of MoRF properties. The disordered regions may fluctuate between several states including coil-like states, localized secondary structure and more compact states. The structural characteristics and the individual states of conformation are determined by the nature of amino acids in the disordered sequences. Thus, to this end, we predicted the structural properties of the disordered region using the structural predictor [16] and utilized it to identify the MoRFs.

To predict amino acid residues of the protein sequence as MoRF and non-MoRF, a learning algorithm requires information of the residue itself and the information of the neighboring residues. However, to predict the terminal residues of the disordered protein sequence, complete neighboring information is not available and this adds complexity to the learning algorithm if a single model is trained to predict all the amino acids of the protein sequence. Therefore, we believe that if separate models are trained to predict the middle and the terminal regions, the performance is thought to improve as the neighboring information of the residues is appropriately incorporated for prediction.

In this paper, we present a MoRF prediction scheme which involves support vector machine (SVM) models to predict MoRFs in protein sequences. In the proposed scheme, separate SVM models are used to predict the terminal and middle regions of a protein sequence. To do this, we have constructed two SVM models, the first one is trained using the terminal regions of training sequences and the second SVM model is trained using the middle region of training sequences. The presented scheme is different from the design approach of other state-of-the-art methods as here separate models are used to predict terminal and middle regions. To complement information present in the protein regions, we followed a similar approach as presented in Malhis et al., [12, 13] and Sharma et al., [15] where scores of many MoRF prediction models are combined. Therefore, we selected the following predictors MoRFpred-plus [14], PROMIS [15] and MoRFchibi [11], and combined their scores with the scores of the proposed model. The main aim of this amalgamation is the use of different sources of information encoded in the protein regions, as this has been proved to improve the MoRF prediction accuracies. The proposed model uses structural information, MoRFpred-plus uses evolutionary profiles and physicochemical properties, MoRFchibi uses physicochemical properties, PROMIS uses structural information and all are developed using a different learning algorithm. The reported performance of the combined model in this study is closer to the benchmarked predictor.

## Method

### Benchmark dataset

To gauge MoRF predictors, in recent studies [8, 11–15], MoRF datasets have been introduced to train and test a model. Table 1 shows the details of these datasets. The datasets TRAIN, TEST, and NEW were collected and assembled by Disfani et al., [8]. To assemble these sets, they collected and filtered the sequence from PDB depositions made before April 2008. The sequences were from different species. They filtered these sets such that each sequence in the set contains MoRF of size between 5 and 25 residues, and sequences in the TEST and NEW sets share less than 30% identity to the sequences in the TRAIN set. The TRAIN set is used to train the proposed model, the TEST set is used to evaluate the model, and we further combine TEST and NEW (as done in previous studies) sets and referred as TEST464 set to compare the MoRF predictors. We found that 42% of the sequences in the TEST464 set share 30% or more sequence identity to one another sequences in the same set. To address this, in our previous work [15] we have filtered the TEST464 set and obtained a resulting set as TEST266 containing 266 sequences. This set is also used for comparison. To validate MoRF predictors, it is important to have test sequences with MoRFs that are verified to be disordered in isolation. However, according to the sequences selection procedure described in Disfani et al., [8], it is not verified that the identified MoRFs in the sequences are disordered in isolation. Therefore, to address the aforementioned issue, we use the dataset EXP53 introduced in Malhis et al., [13] to report the performance. EXP53 contains 53 non-redundant protein sequences that have MoRFs experimentally validated to be disordered in isolation.

**Table 1:** Datasets used to train and test a MoRF predictor

Data sets		No. of Sequences	Total residues	No. of MoRF residues	No. of non-MoRF residues
training set	TRAIN	421	245,984	5396	240,588
test sets	TEST	419	258,829	5153	253,676
	NEW	45	37,533	626	36,907
	TEST464	464	296,362	5779	290, 583
	TEST266	266	154,399	3305	151,094
validation set	EXP53	53	25,186	2432	22,754

### Overview of the proposed method

**Fig. 1:**
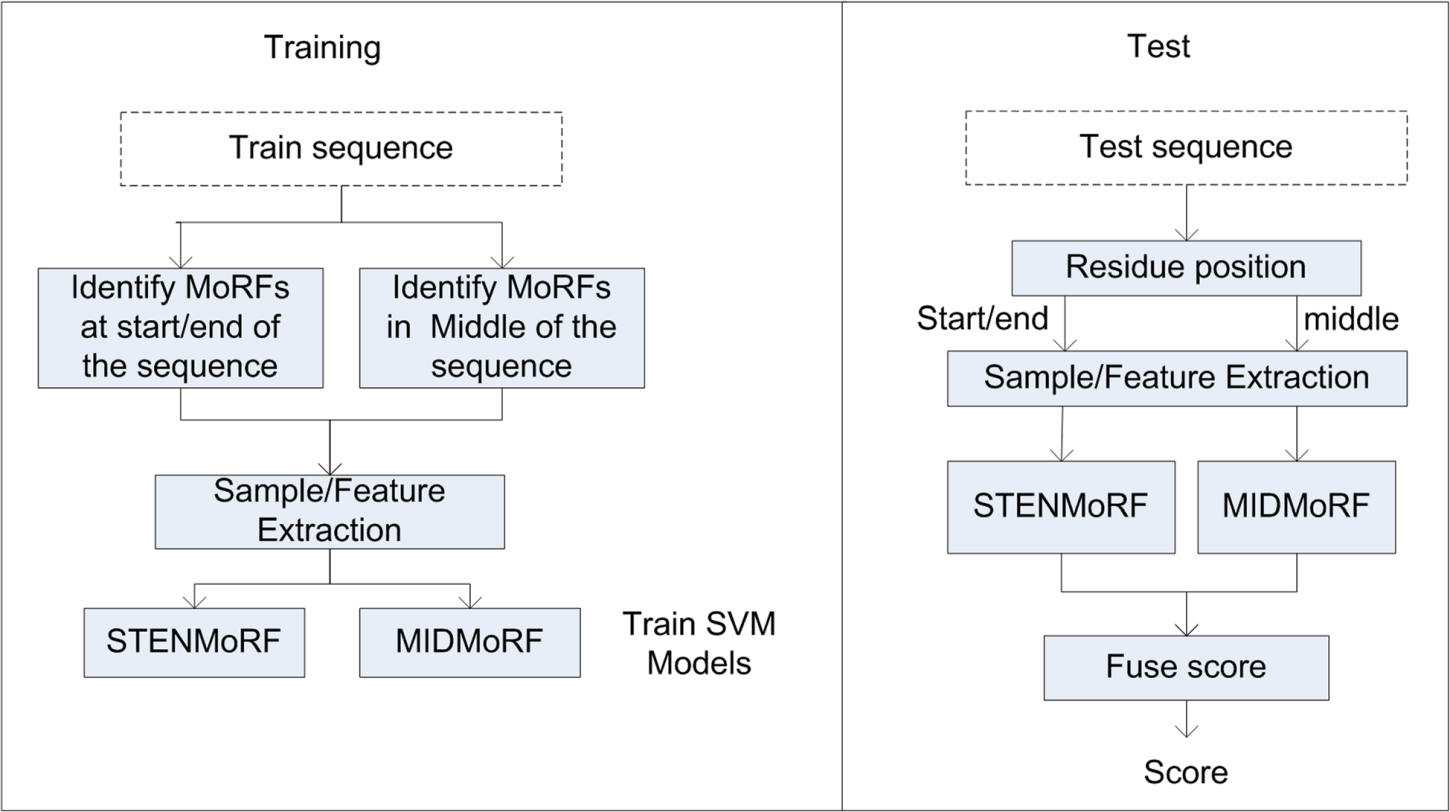
Overview of the proposed method. Fuse score means that the model scores are combined to provide the whole sequence scores

To predict residues of intrinsically disordered protein sequences as MoRF or non-MoRF, a machine learning algorithm requires information of the residue itself and the information of the neighboring residues. However, to predict terminal residues of the disordered protein sequence, complete neighboring information is not available and this adds complexity to the learning algorithm to correctly predict MoRFs. To overcome this problem, in this study, we trisect the disordered protein sequence into the terminals and middle regions and we train two different models to predict these regions. Figure 1 shows the overview of the proposed method. The first model (STENMoRF) is used to predict the terminal regions of the protein sequences and the second model (MIDMoRF) is used to predict the middle region of the protein sequences. To incorporate structural information, we computed features using backbone torsion angles, secondary structure (SS), half-sphere exposure (HSE) and accessible surface area (ASA) of the disordered protein sequence.

There exist many tools to obtain structural information of a protein sequence. In this study, we utilized SPIDER2 predictor [16] to predict the structural attributes such as SS, ASA, HSE and backbone torsion angles of the protein sequences. SS represents the structural description of the protein sequence in a number of discrete states, such as helix, coil, and sheet. SS output is a three-dimensional vector containing the transition probabilities to three secondary structures. ASA represents the exposure level of the amino acids to solvent in a protein sequence and the output is a one–dimensional vector representing the structural property. Backbone angles contain the backbone dihedral angles of the amino acids in the protein sequence. These angles are Phi, Psi, Theta (*θ*) and Tau (τ). HSE provides the number of C alpha atoms in the upper and lower spheres of the amino acids. We used the measures including HSE alpha and HSE beta along with the contact numbers for the amino acids.

### Support vector machine

An SVM classifier with radial basis function (RBF) is used for MoRF prediction. We have used the same values of C and gamma (1000 and 0.0038) as in our previous study [15] to evaluate the proposed method. We have selected these values because the datasets used and features computed in both studies are similar and also these values provided good results in our previous study [15].

### Training

In the training step, we extract features from MoRFs and non-MoRFs. Suppose a protein sequence *P*_*i*_ is given as:

1 $$ {P}_i={A}_1{A}_2\dots {A}_j\dots {A}_{n_i}\kern1em \left(i=1,2,\dots, T\right) $$

where *A*_*j*_ is the *j*-th amino acid in the sequence, *T* is the total number of protein sequences in the training set and *n*_*i*_ is the length of protein sequence *P*_*i*_. Before we define the positive and negative segments representing MoRFs and non-MoRFs, it is essential to select a suitable flank size (the length of neighboring residues), as this size will determine the length of the terminal regions. We selected the flank size as 20 from our previous study [15] because this flank size provided good performance for MoRF prediction. Using flank size as 20, the segments were extracted in the following way: suppose for a protein *P*_*i*_ if the *j*-th amino acid is part of MoRF region for 1 ≤ *j* ≤ 20 and *n*_*i*_ − 20 < *j* ≤ *n*_*i*_, we extract the MoRF region plus flank regions of 20 amino acids upstream and downstream (if exist) of MoRF region as a positive segment for STENMoRF; and, if *j*-th amino acid is a part of MoRF region for 20 < *j* ≤ *n*_*i*_ − 20, we extract the MoRF region plus flank of 20 amino acids upstream and downstream of MoRF region as a positive segment for MIDMoRF. Besides, a negative segment (same size as a positive segment) is extracted from a non-MoRF region in a similar way for STENMoRF and MIDMoRF, respectively.

We extract an equal number of positive and negative samples using the steps of the StructMoRF method described in Sharma et al., [15], i.e., positive sample is extracted from a positive segment and negative sample is extracted from a negative segment, and to compute the feature vector for the samples, we used structural attributes. Suppose if the *u*-th number of the attribute is considered, the structural matrix *M* for a sample *S* of length *l* will be given as:

2 $$ M=\left[\begin{array}{cccc}{M}_{1,1}& {M}_{1,2}& \cdots & {M}_{1,u}\\ {}{M}_{2,1}& {M}_{2,2}& \cdots & {M}_{2,u}\\ {}\vdots & \vdots & \ddots & \vdots \\ {}{M}_{l,1}& {M}_{l,2}& \cdots & {M}_{l,u}\end{array}\right] $$

where *M*_*i*, *j*_ is the element of a matrix *M* for 1 ≤ *i* ≤ *l and* 1 ≤ *j* ≤ *u*. To extract features from matrix *M*, we use auto-covariance based features for STENMoRF. Auto-covariance feature is computed from matrix *M* as follows:

3 $$ {AC}_{k,j}=\frac{1}{l}\ \sum \limits_{i=1}^{l-k}{M}_{i,j}\ {M}_{i+k,j}\ \left(j=1,\dots .,u\ \mathrm{and}\ k=1\dots DF\right) $$

where *DF* is the distance factor. The computed feature matrix *AC*_*k*, *j*_ will be of size *DF* × *u* and can be rearranged in a vector form by reshaping it into a vector of length *DF* × *u*. Observing the performance, the effective value of *DF* was obtained as 10. Moreover, to extract features for MIDMoRF, we use feature extraction procedure of structMoRF method described in Sharma et al., [15].

### Test

To score each residue in the query protein sequence, we extract a sample for each query residue using the window of size 41 (flank size× 2 + 1). Except for the terminal region residues, the sample length will be of 41 amino acids. For a query residue, sample *S*_*j*_ is defined as

4 $$ {S}_j=\left\{\begin{array}{c}\ {A}_1,{A}_2,{A}_3\cdots \cdots \cdots, {A}_{j+20},\kern19.5em j\le 20\kern0.5em \\ {}{A}_{j-20},\kern0.5em \cdots \cdots \cdots {A}_{L-2},{A}_{L-1},{A}_L,\kern15.75em j>L-20\\ {}\ {A}_{j-20},\kern0.5em \cdots \cdots \cdots {A}_{j-2},{A}_{j-1},{A}_j,{A}_{j+1},{A}_{j+2}\cdots \cdots \cdots, {A}_{j+20},\kern3.5em otherwise\end{array}\right.\kern0.75em $$

where *A*_*j*_ is the query residue in the query sequence, *j*=1,2,...*L* and *L* is the length of the query protein sequence. Samples for a query sequence of length *L* can be is interpreted using eq. (4) as:

5 $$ {\gamma}_{ts}=\left\{\begin{array}{c}\kern0.5em {S}_{1\kern1em }\\ {}{S}_2\\ {}\vdots \\ {}\vdots \\ {}\vdots \\ {}{S}_L\end{array}\right. $$

**Fig. 2:**
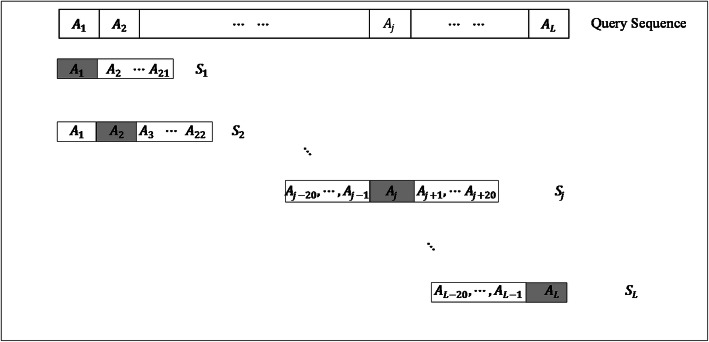
Schematic illustration of extracting samples to score a query sequence. ***A***_***j***_ is the *j*-th amino acid in the query sequence and ***L*** refers to the length of the query protein sequence

Figure 2 shows the schematic illustration of extracting query samples from a query protein sequence. First 20 and last 20 samples representing terminal region residues are scored using STENMoRF and the remaining samples are scored using MIDMoRF.

### Performance measure

We use the performance measures AUC, true positive rate (TPR) and false positive rate (FPR) to evaluate the models in this study, where AUC is defined as the area under the receiver characteristics curve.

### Combined model

**Fig. 3:**
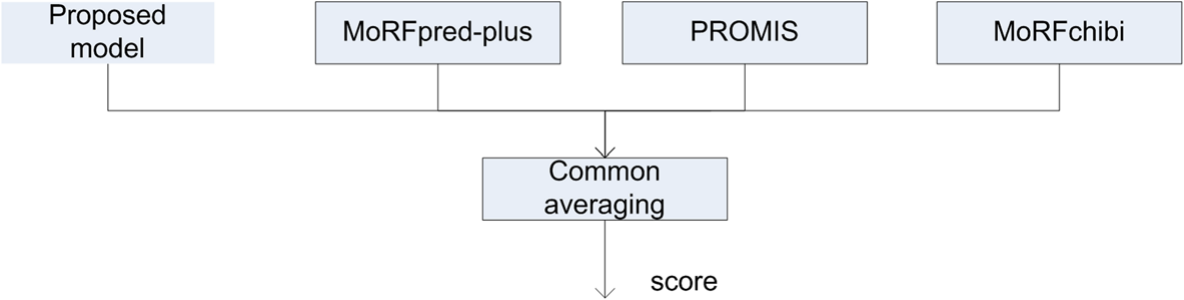
Combined model. MoRFpred-plus and PROMIS are our predictors while we download MoRFchibi predictor and integrate it with our proposed model

The proposed model predicts the terminal and middle regions of the disordered protein sequence by incorporating structural information. According to the previous studies [12, 13, 15], combining different learning algorithms with different sources of information is supposed to provide more information for MoRF prediction. Thus, we selected the recently published MoRF predictors (MoRFpred-plus [14], PROMIS [15] and MoRFchibi [11]) and combined their output scores with the scores of the proposed model. Figure 3 shows the details of the combined scheme. To combine the output scores, we apply the common averaging principle, where scores of all the models are averaged.

## Results

The performance in this study is reported using the same datasets that were used to analyses MoRF predictors such as MoRFchibi, MoRFpred, MoRFpred-plus, MoRFchibi-web, and OPAL. In this section, we present the model tuning scheme followed by the performance comparison.

### Model tuning

Feature selection techniques are very crucial for machine learning algorithms, as it reduces the computational complexity of the algorithm by reducing the feature dimension and it also selects best features to represent the data. In this study, we used successive feature selection scheme in the forward direction [17] to choose structural attributes for each of the model. Evaluating the scheme using structural attributes, the proposed models provided good performance (AUCs) with attributes from half-sphere exposure (HSE) *α* and *β* group. HSE is a measure of solvent exposure of a residue and it gives the number of C alpha atoms in the upper and lower spheres [18]. As more structural attributes are concatenated using the scheme, the performance deteriorates. Therefore, we used the attribute HSEu from the HSE *α* group to extract features for the proposed models.

**Fig. 4:**
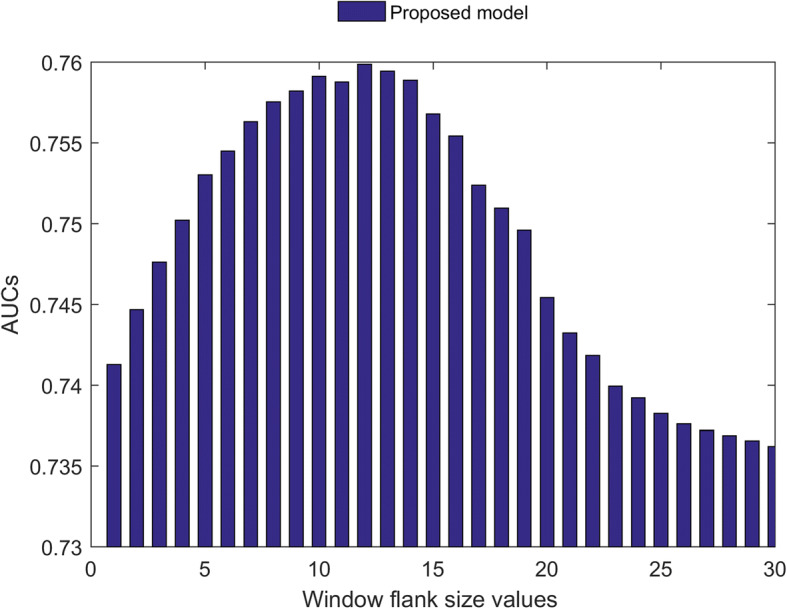
AUCs for the proposed model with varying window flank size values to process the output scores

Furthermore, MoRFs considered in this study are of a size greater than 5 residues. Therefore, a query residue predicted as MoRF should be a part of MoRF region. To incorporate this criterion into the proposed scheme, we used the score calculation technique from our previous study [15] to process and compute the output scores of each model used in the combined scheme of Fig. 3. The procedure of processing the scores involved the following steps: (1) take the window of scores for each residue, i.e., residue score plus region of flank scores on both sides; (2) compute the final score as the maximum of the window scores plus the median of the window scores divided by two. Thus, for each of the model, we varied the window flank size values from 1 to 30 to process the output scores, and we selected the best window flank size value for each model by observing the AUC performance measure. From Fig. 4, we note that the proposed model performs well at window flank size value of 12 and to get average performance from MoRFpred-plus, MoRFchibi and combined proposed model, we processed their output scores with window flank size values of 4, 15 and 8, respectively. To show the increase in performance using separate models, instead of a single model used to predict the entire sequence, (Additional file 1: Table S1) describes the performance.

### Performance comparison

We reported AUCs using the datasets TEST, TEST464, TEST266, and EXP53. The datasets TEST, TEST464 and TEST266 contain sequences with MoRFs of length 5 to 25 amino acids. However, sequences in the EXP53 dataset include MoRFs of length greater than 30 amino acids. Therefore, we report the performance of EXP53 as EXP53 ALL (contains all MoRFs), EXP53 SHORT (contains MoRFs up to the length of 30 amino acids) and EXP53 LONG (contains MoRF greater than 30 amino acids in length). Table 2 shows the performance of the proposed and combined models. Although the models are trained to predict short MoRFs, we also reported performance for long MoRFs to see how the models perform while predicting long MoRFs. As observed in Table 2, the proposed combined model performed similar to the benchmarked OPAL predictor. Hence, the novelty in this study is that we have presented a new alternative method of MoRF prediction and have also obtained close results compared with the state-of-the-art predictors.

**Table 2:** AUCs using the test sets

Predictors/models	TEST	TEST464	TEST266	EXP53 ALL	EXP53 LONG	EXP53 SHORT
ANCHOR	0.6	0.605	0.599	0.615	0.586	0.683
MoRFpred	0.673	0.675	0.651	0.62	0.598	0.673
MoRFchibi	0.74	0.743	0.709	0.712	0.679	0.79
MoRFpred-plus	0.755	0.724	0.740	0.712	0.67	0.821
MoRFchibi-light	0.775	0.777	0.762	0.799	0.77	0.869
PROMIS	0.791	0.788	0.770	0.818	0.815	0.823
MoRFchibi-web	0.8	0.805	0.785	0.797	0.758	0.886
OPAL	0.815	0.816	0.795	0.836	0.823	0.870
Proposed Model	0.760	0.757	0.729	0.787	0.754	0.864
Combined Model	0.819	0.818	0.797	0.838	0.819	0.881

We further evaluated the performance of the proposed model against the benchmarked OPAL predictor. For comparison, we plotted the propensity score of proteins P15337, P26645, P02686, P42768 and Q99967 from the EXP53 set. (Additional file 1: Figures S1 to S5) shows the propensity scores for each of the protein. We particularly observe that where OPAL performs poorly, the proposed model upgrades the scores of the verified MoRF regions. The analysis also showed that for some non-MoRF residues, the propensity scores of the proposed model are lower compared with that of OPAL.

In detail, comparing the proposed method with MoRFchibi-web and OPAL, we obtained performance improvement (in terms of AUCs) of 1.9% and 0.4% using TEST set, 1.3% and 0.2% using TEST464 set, 1.2% and 0.2% using TEST266 set, and 4.1% and 0.2% using EXP53 ALL set, respectively. Furthermore, we observe that OPAL performed better in predicting long MoRFs, whereas MoRFchibi-web obtained good performance in scoring short MoRFs. Thus, on an average scale, the proposed method has boosted the performance of scoring short MoRFs by 1.1% compared to OPAL.

## Discussion

**Fig. 5:**
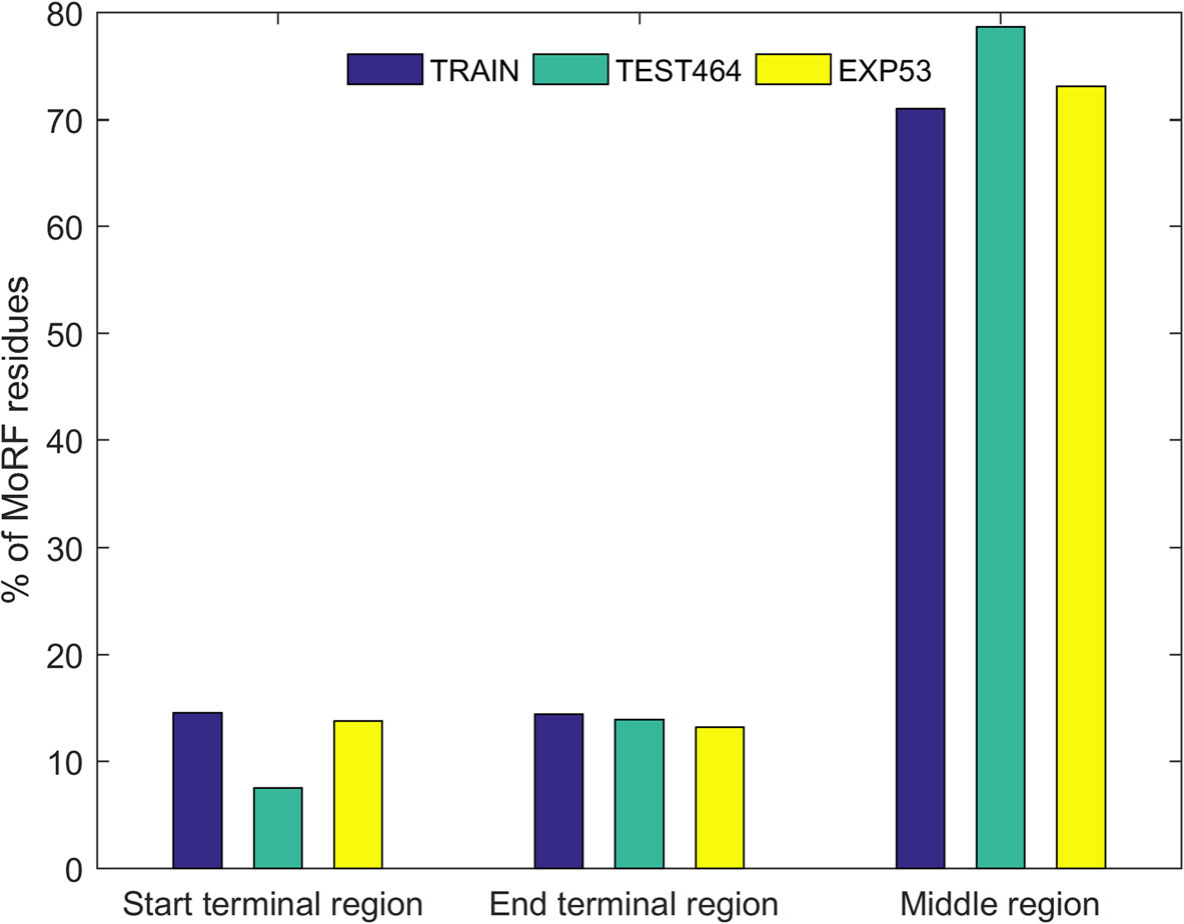
Percentage of MoRFs present in terminal and middle regions

In this study, we presented the method of identifying MoRFs in disordered protein sequences. The method involves the construction of two SVM models, the first model is used to predict the terminal regions and the second model is used to predict the middle region of the disordered protein sequences. We decided to construct separate models for the two following reasons. First, since the residues in the middle region contain full neighboring information whereas the residues in the terminal regions do not contain full neighboring information, therefore, if a single model is to be used to predict both the regions, then complexity is added in identifying the MoRF residues. Second, MoRF regions in the datasets are distributed on the entire protein sequences, i.e., we note that in the TEST464 set, there are 296,362 residues and out of this 18,560 residues in this study are considered as terminal regions with 30% of which are MoRF residues. Therefore, it is necessary to score such a large number of terminal residues using a separate model to avoid fault detection of MoRFs. Figure 5 shows the percentage of MoRF residues present in the terminal and middle regions of the TRAIN, TEST464 and EXP53 sets.

The sequences in the TRAIN set contain MoRFs of variable size from 5 to 25 residues, and a single MoRF is present per sequence. Thus, this brings the issue of unbiased data, as the number of non-MoRF residues is more significant compared to the number of MoRF residues. To overcome this issue, during training step we have selected positive samples from MoRFs and we have extracted the same number of negative samples from non-MoRFs.

**Fig. 6:**
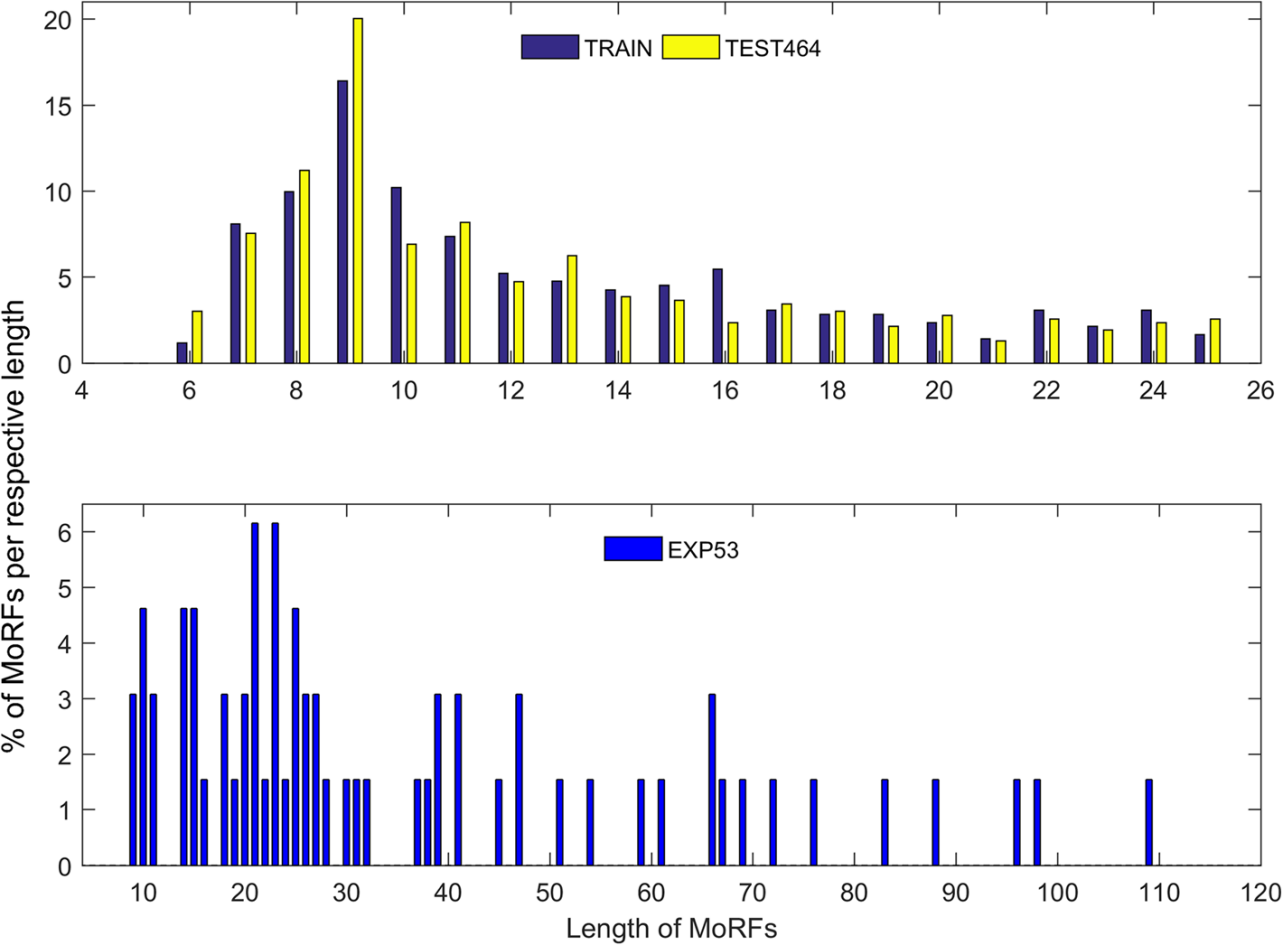
Percentage of MoRFs per respective length for the TRAIN, TEST464 and EXP53 sets

To perform analyses on the average length of MoRFs used for training and evaluation, we plotted the number of MoRFs available for each length. Figure 6 shows the analyses of MoRFs for the TRAIN, TEST464 and EXP53 sets. For the TRAIN and TEST464 sets, a larger number of the MoRFs are of length 7 to 11 residues while an equal number of MoRFs are present for the other lengths. The EXP53 set contains short and long MoRFs, and thus in Fig. 6, it is observed that more MoRFs are present for length 10 to 28 residues while less number of MoRFs are present for length 29 to 110 residues. Since the models are trained using short MoRFs, to evaluate EXP53 set, we report the performance for EXP53 short MoRFs up to 30 residues and in addition to see how the models perform in predicting long MoRFs greater than 30 residues in length, we reported performance for EXP53 long MoRFs separately. The models show good results for predicting short MoRFs, and even though the models were trained to predict short MoRFs, they performed well in scoring long MoRFs. This was achievable because the models use residue information and its upstream/downstream neighboring residue information for prediction.

The comparable performance obtained by the proposed combined model in comparison with the benchmarked state-of-the-art predictors achieved due to the following implementation:

1. use of different sources of information of disordered regions such as structural attributes; evolutionary profiles, and physicochemical attributes.
2. use of different learning algorithms obtained by combining scores of the proposed model with the scores of MoRFpred-plus, PROMIS and MoRFchibi.
3. selecting an equal number of positive and negative training samples from unbiased MoRF and non-MoRF regions.
4. processing output scores, this processing provided extra information to see if the neighboring residues have high scores to form a MoRF region or not.

Incorporating each of the mentioned implementation, the complementary information residing in the protein regions were extracted and combined for MoRF prediction. To compare the combined model with the benchmarked OPAL predictor, Table 3 shows the FPR values for a range of TPR values. Thus, similar performance is noted.

**Table 3:** FPR for a given TPR value for the combined model and OPAL using EXP53 SHORT

TPR	0.2	0.3	0.4	0.5	0.6	0.7	0.8	0.9
OPAL	0.0113	0.0158	0.0414	0.0691	0.0902	0.1144	0.216	0.334
Combined model	0.0118	0.0175	0.0323	0.0593	0.0889	0.1150	0.1852	0.2913

## Conclusion

In this study, disordered protein sequences are trisected into the terminal and middle regions for MoRF prediction. Incorporating structural, evolutionary and physicochemical information of disordered proteins, a comparable performance is achieved compared with the performance of the state-of-the-art MoRF predictors. Thus, the proposed method can be used as an alternative approach for MoRF prediction.

## Additional file

Additional file 1: Supplementary text for Discovering MoRFs by trisecting intrinsically disordered protein sequence into terminals and middle regions. (PDF 446 kb)

## Abbreviations

ASA: Accessible surface area
AUC: Area under the curve
FPR: false positive rate
HSE: Half-sphere exposure
IDPs: Intrinsically disordered proteins
IDRs: Intrinsically disordered regions
MoRFs: Molecular recognition features
RBF: Radial basis function
SS: Secondary structure
SVM: Support vector machine
TPR: True positive rate
