# Supplementary material for: Discovering MoRFs by trisecting intrinsically disordered protein sequence into terminals and middle regions
Source: BMC Bioinformatics. 2019 Feb 4;19(Suppl 13):378. doi: 10.1186/s12859-018-2396-7 (PMC7653905; doi:10.1186/s12859-018-2396-7)
Supplement: Supplementary file 1 — Supplementary text for Discovering MoRFs by trisecting intrinsically disordered protein sequence into terminals and middle regions. (PDF 446 kb) [file 12859_2018_2396_MOESM1_ESM.pdf]

Additional File 1: Supplementary text for Discovering MoRFs by trisecting intrinsically disordered protein sequence into terminals and middle regions

In this supplementary text, we have evaluated the performance of the proposed model against the benchmarked OPAL predictor. For comparison, we have plotted the propensity scores of 5 proteins (P15337, P26645, P02686, P42768 and Q99967) from the EXP53 set. Figures S1 to S5 shows the propensity scores for each of the protein, the scores are given by the proposed model in blue and OPAL in red. The verified section of the MoRFs in each of the protein is marked in yellow color.

Furthermore, MoRF protein has 3 regions of information, middle region, left terminal and right terminal. Therefore, considering these regions for analysis would be appropriate. Table S1 shows the results for four cases of analyzing these regions. These cases are as follows:

Case 1: one model trained to predict the entire sequence alone.
Case 2: two models trained, first one to predict the terminal regions, and the second one to predict the middle region.
Case 3: three models trained, first one to predict the left terminal region, second one to predict the right terminal region and the third one to predict the middle region.
Case 4: 4 models trained, first one to predict the left terminal region, second one to predict the right terminal region, the third one to predict the first half of the middle region and the fourth one to predict the second half of the middle region.

Observing the results in Table S1, we note that as the number of segments and models increase the performance deteriorates. Thus, we select case 2, where the protein sequence is trisected and two models are trained to predict the terminal and the middle regions.

Figure S1: Propensity scores for protein >P15337|1KDX_B|m118-145. Compared with OPAL, the proposed model obtains higher scores at the verified MoRF region. MoRF location is marked in yellow color.

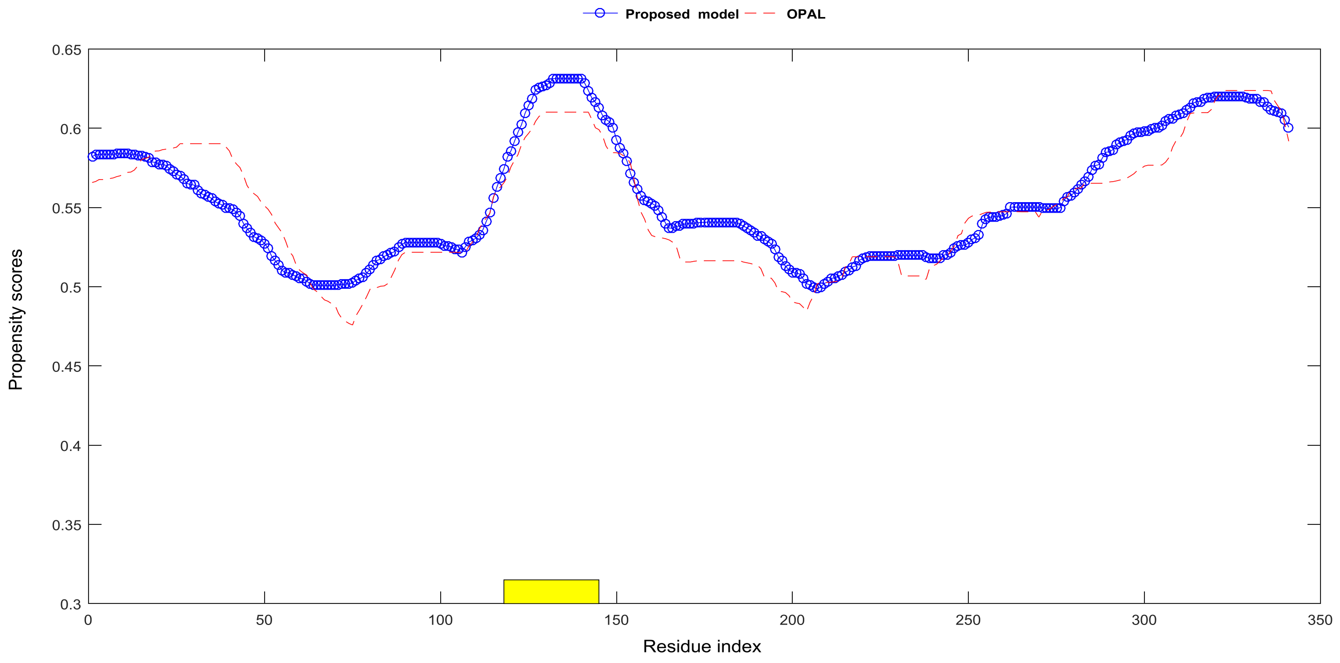

Figure S2: Propensity scores for protein >P26645|1IWQ_B|m147-164. Compared with OPAL, the proposed model obtains higher scores at the verified MoRF region. It also lowers the scores at the end terminal residues, where MoRFs do not exist. MoRF location is marked in yellow color.

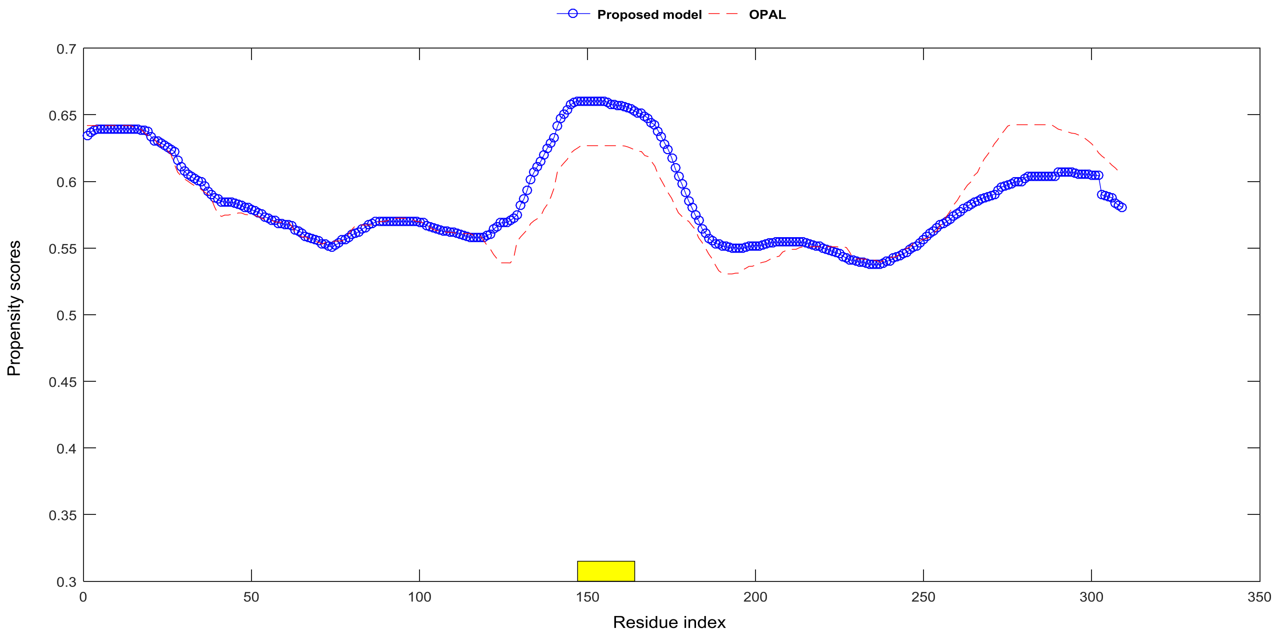

Figure S3: Propensity scores for protein >P02686-5|1K2D_P|1FV1_C|1YMM_C|E8|m1-11|m83-103|m145-165. The scores of the proposed model are very similar to OPAL. Small increases in the score values are noted for MoRFs between residues 83 and 103. MoRF location is marked in yellow color.

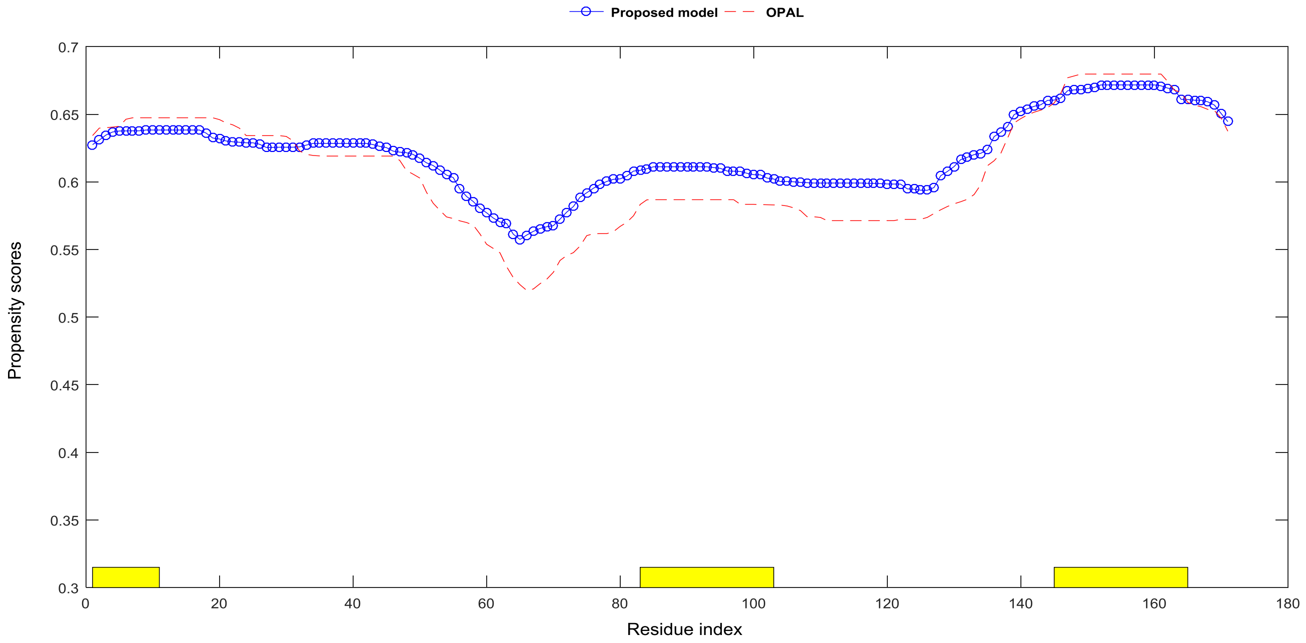

Figure S4: Propensity scores for protein >sp|P42768|1T84|1CEE|M229-287|M460-491. Observing the propensity scores of the proposed model, it is believed that MoRFs are detected more accurately compared to OPAL. MoRF location is marked in yellow color.

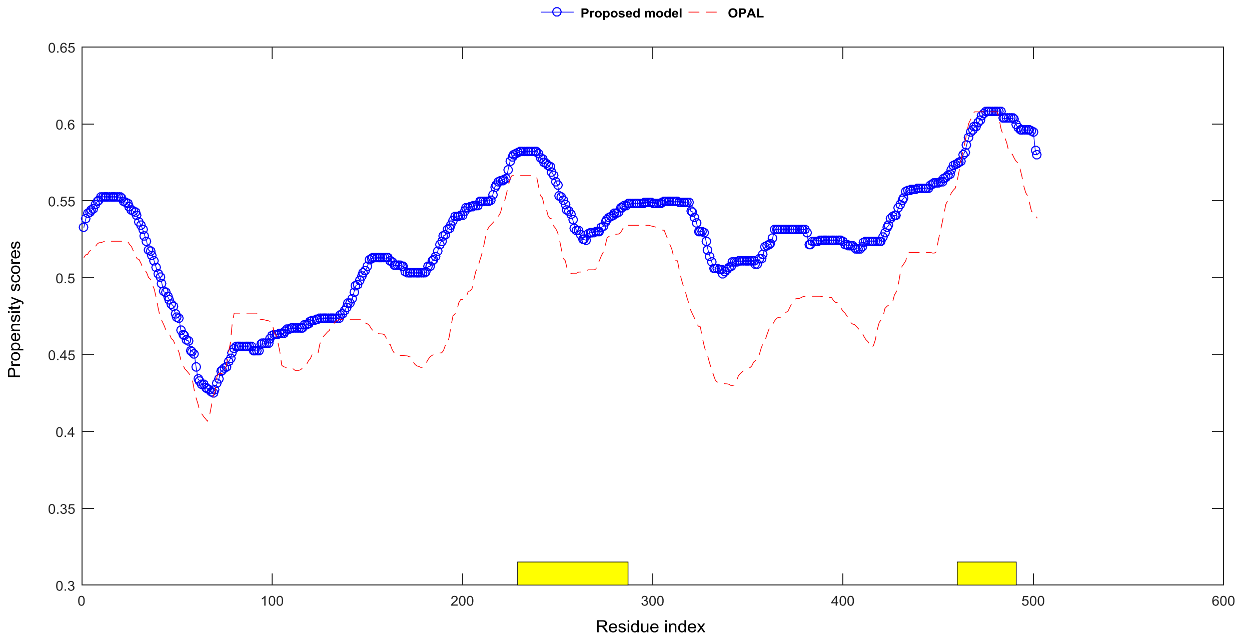

Figure S5: Propensity scores for protein >Q99967|1P4Q_A|1R8U_A|M215-268. The verified MoRF region is detected more accurately by the proposed model. It is also noted that OPAL produces higher scores at the start terminal residues where MoRFs do not exist, but with the proposed model, the scores are lower. MoRF location is marked in yellow color.

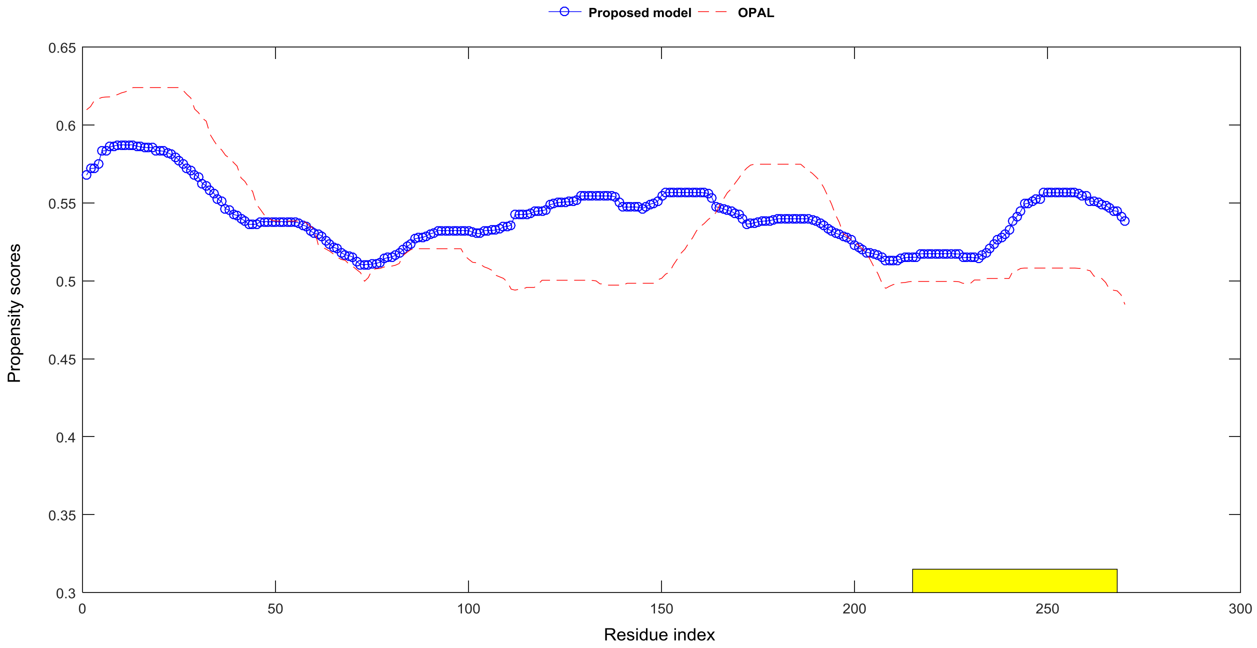

Table S1: AUCs are based on the number of segments and models used. Initially, using the single model for prediction, we selected flank size of 20 and performed successive feature selection scheme in the forward direction to select best performing attributes. Extracting features from these attributes, we trained the models based on the number of segments created. These models are then used to score the TEST data and AUCs are reported.

Case	Number of segments	Number of models	AUCs
1	1	1	0.755
**2**	**3**	**2**	**0.760**
3	3	3	0.741
4	4	4	0.705
